# Supplementary material for: Synthetic BZLF1-targeted transcriptional activator for efficient lytic induction therapy against EBV-associated epithelial cancers
Source: Nat Commun. 2024 May 3;15:3729. doi: 10.1038/s41467-024-48031-8 (PMC11068728; doi:10.1038/s41467-024-48031-8)
Supplement: Supplementary file 1 — Supplementary Information [file 41467_2024_48031_MOESM1_ESM.pdf]

Synthetic *BZLF1*-targeted transcriptional activator for efficient lytic induction therapy against EBV-associated epithelial cancers

Man Wu, Pok-Man Hau, Linxian Li, Chi Man Tsang, Yike Yang, Aziz Taghbalout, Grace TY Chung, Shin Yee Hui, Wing Chung Tang, Nathaniel Jillette, Jacqueline Jufen Zhu, Horace Hok Yeung Lee, Ee Ling Kong, Melissa Sue Ann Chan, Jason YK Chan, Brigette BY Ma, Mei-Ru Chen[9], Charles Lee, Ka Fai To, Albert Wu Cheng, Kwok-Wai Lo

Supplementary information

Supplementary Figure 1

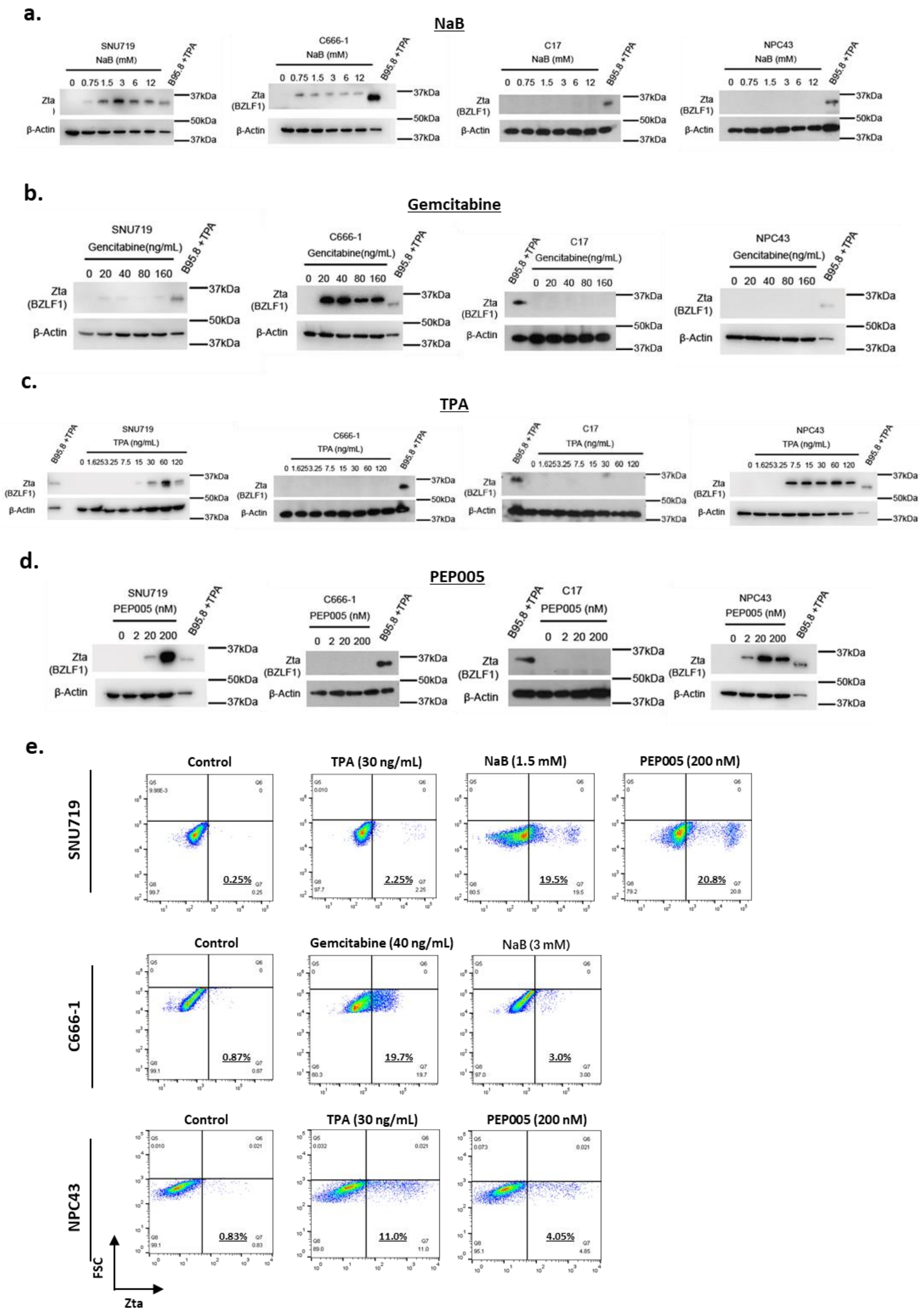

Supplementary Figure 1

Supplementary Figure 1. Zta expression in EBV-positive cancer cells treated with chemical inducers. Using western blotting, Zta expression was examined in EBVaGC (SNU719 and NPC (C666-1, NPC43 and C17) cell lines after treatment with chemical inducers including **(a)** NaB, **(b)** gemcitabine, **(c)**TPA, and **(d)** PEP 005 (n = 3 experimental replicates). The TPA-treated EBV-positive lymphoblastoid cell line B95-8 was included as a control for Zta expression. None of the chemical inducer successfully induced Zta expression in C17 cells. **e,** The efficiency of Zta induction in EBV-positive epithelial cells treated with chemical inducers was determined using flow cytometry. A representative flow cytometry analysis and the percentages of Zta-expressing SNU719, C666-1 and NPC43 cells treated with various chemical inducers are shown (n = 3 experimental replicates). Source data are provided as a Source Data file.

Supplementary Figure 2

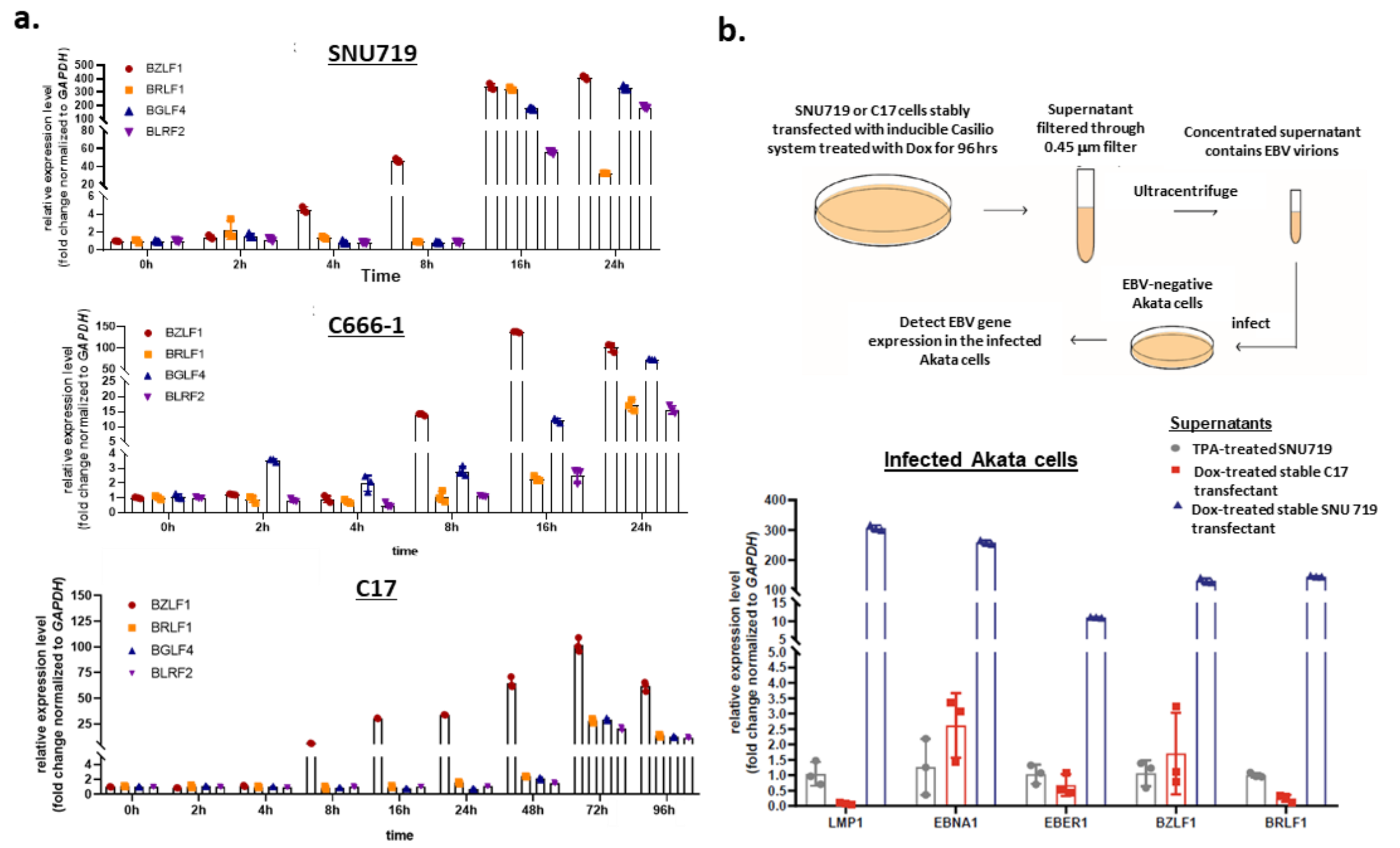

Supplementary Figure 2. Activation of *BZLF1* in EBV-positive epithelial cancer cells stably transfected with the inducible Casilio activator system. a, Quantitative RT-PCR analysis revealed the expression of *BZLF1*, *BRLF1*, *BGLF4* and *BLRF2* in SNU719, C666-1 and C17 cells stably transfected with sgRNA3, HA-dCas9-EGFP and the inducible 3xFLAG-PUFa-p65HSF1 transactivator at 0, 2, 4, 8,16, and 24 h (or 0, 16, 24, 48, 72 and 96 h) after Dox treatment (n = 3 experimental replicates). Data are presented as mean ± SD. **b,** Production of infectious EBV virions in C17 and SNU-719 cells stably transfected with sgRNA3, HA-dCas9-2A-EGFP and inducible 3xFLAG-PUFa-p65HSF1 transactivator after 96 h of Dox treatment. EBV negative Akata cells were cultured with the supernatants collected from Dox-treated C17 and SNU719 stable transfectants. The supernatant from TPA-treated SNU719 cells was used as a control. Using quantitative RT PCR, the expression levels of EBV-encoded latent (*LMP1, EBNA1,* and *EBER1*) and lytic (*BZLF1* and *BRLF1*) genes in infected Akata cells were determined (n = 3 experimental replicates). Data are presented as mean ± SD. Source data are provided as a Source Data file.

Supplementary Figure 3

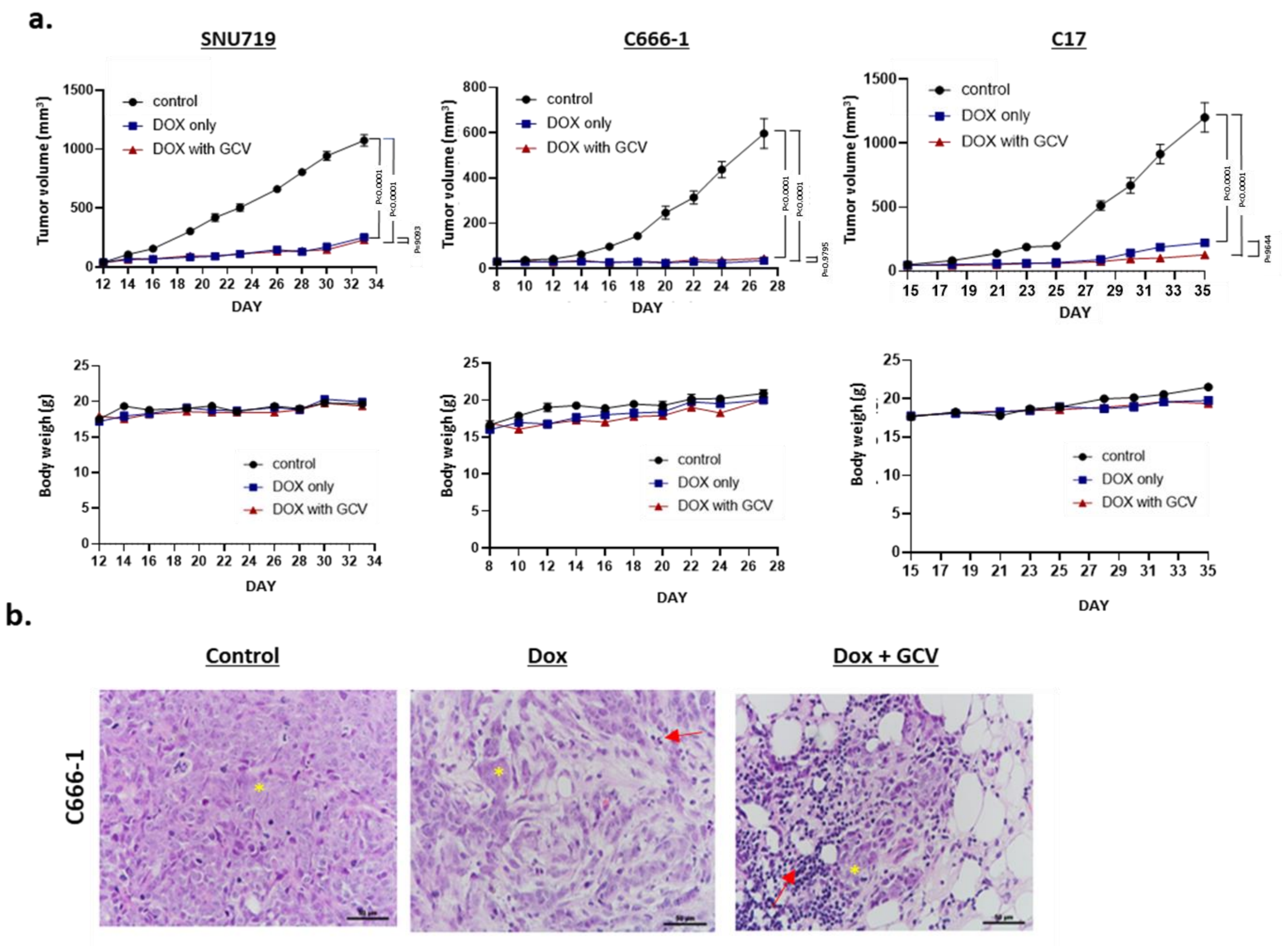

Supplementary Figure 3. ***In vivo*** **antitumor effects of** ***BZLF1*** **activation in EBV-positive epithelial cancer cells stably transfected with the inducible Casilio activator system.** **a,** The *in vivo* activation of endogenous *BZLF1* resulted in potent antitumor effects on cell-derived xenograft models of SNU719, C666-1 and C17 stable transfectants. To establish tumors derived from SNU719, C666-1 and C17 cells stably transfected with sgRNA3, HA-dCas 9-2A-EGFP and the inducible 3xFLAG-PUFa-p65HSF1 transactivator, $2x10^6$ cells were subcutaneously inoculated into the flanks of 3-4-week-old female athymic mice, and the tumors were allowed to grow to a size of 50 mm^3. Eight mice were allocated to each treatment or control group. The tumor size was measured every 2-3 days after daily treatment with (Dox 625 mg/kg), or a combination of Dox and GCV (10 mg/kg, i.p. daily), or control treatment (n = 8 mice/group). Date are presented as mean ± SD. A P value < 0.05 was considered to indicate statistical significance. One-way analysis of variance (ANOVA). **b,** Representative images of H&E-stained FFPE sections to illustrate the histological features of the residual tumors (*) harvested

from mice treated with Dox or combined Dox and GCV (n = 8 mice/group). Compared with the control, heavy fibroblast and lymphocyte infiltration (red arrows) was observed in the residual tumors after Dox or combined Dox and GCV treatment. Scale bar = 50 μm. Source data are provided as a Source Data file.

Supplementary Figure 4

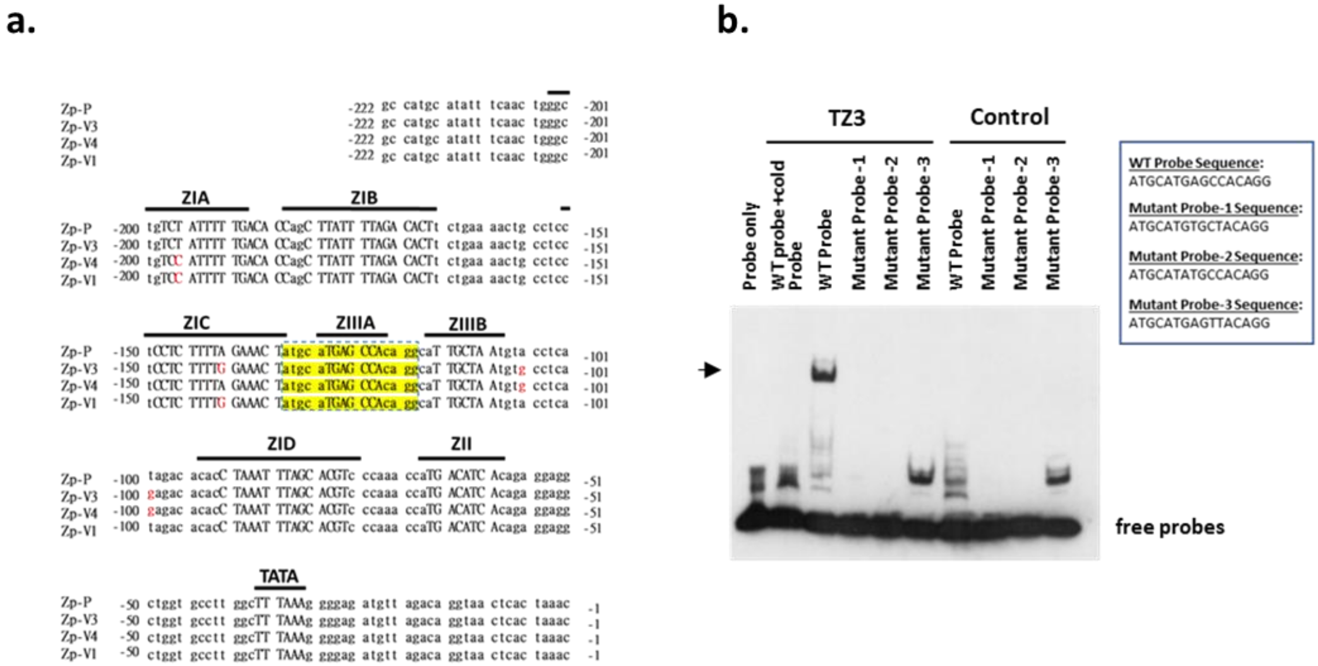

Supplementary Figure 4. Specific binding ability of the TZ3 transcriptional activator to the *BZLF1* promoter. **a,** The promoter sequences of wild-type EBV *BZLF1* (B95-8, Zp-P) and reported variants (Zp-V3, Zp-V4 and Zp-V1) are shown. Cis-regulatory element sequences and sequence variations are shown in capital letters and red font, respectively. The Z3-binding sequence is highlighted in yellow and is conserved among all EBV strains. **b,** Detection of the specific binding ability of the TZ3 transcriptional activator to the targeted sequence in the *BZLF1* promoter in C666-1 cells by EMSA (arrow). In addition to a probe specific for the wild-type (WT) target sequence in the *BZLF1* promoter, three mutant probes were included. Their sequences are shown in the box. Cells transfected with vector alone were used as the control (n = 3 experimental replicates).

Supplementary Figure 5

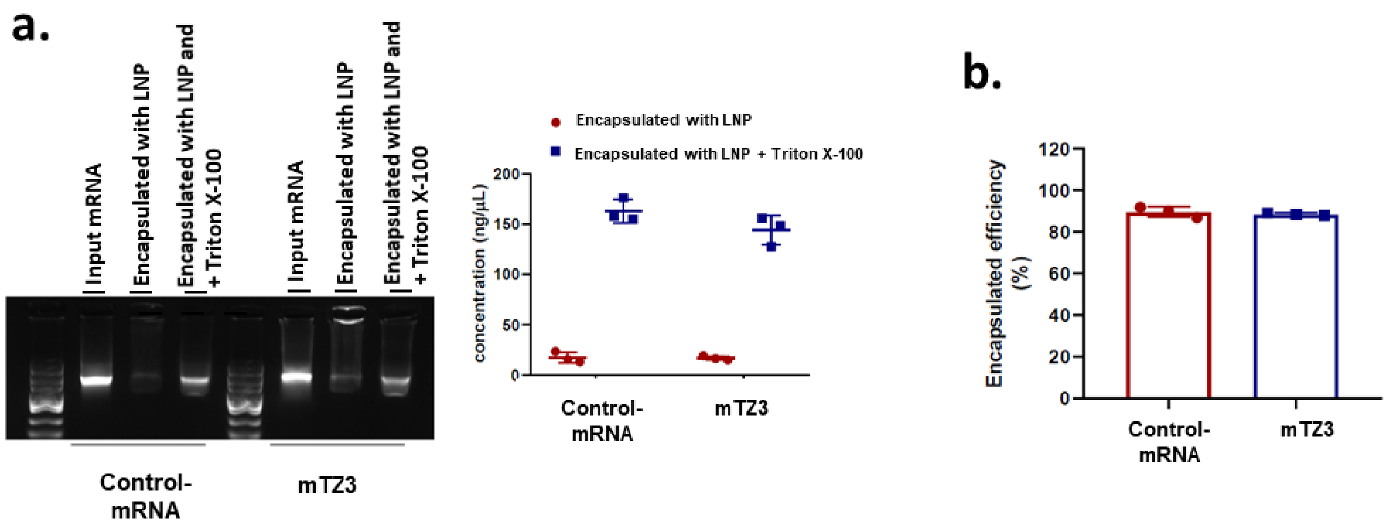

Supplementary Figure 5. mRNA encapsulation efficiency of the formulated LNP. a, To determine the efficiency of mRNA encapsulation using the formulated LNP, the mRNA amount and concentration were measured in the input mRNA, formulated mRNA-LNP (mTZ3 and control mRNAs) and mRNA-LNP with Triton x-100 treatment using gel electrophoresis and a Quanti-iT RiboGreen RNA assay (n = 3 experimental replicates). Data are presented as mean ± SD. **b,** High mRNA encapsulation efficiency was observed for both mTZ3-LNP and control mRNA-LNP (n = 3 independent experiments/group). Data are presented as mean ± SD. Source data are provided as a Source Data file.

Supplementary Figure 6

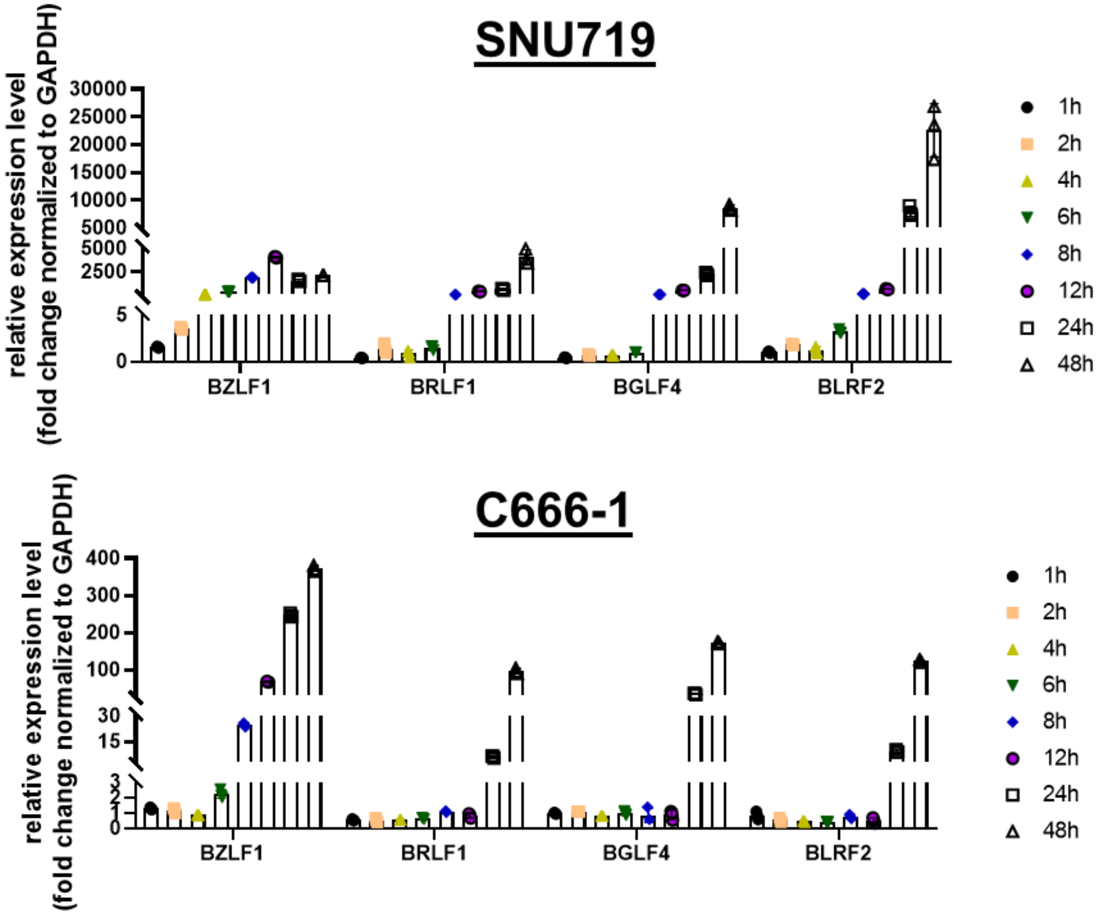

Supplementary Figure 6. mTZ3-LNP treatment reactivated EBV lytic genes in EBV-associated cancers. Quantitative RT-PCR analysis revealed the expression of *BZLF1, BRLF1, BGLF4* and *BLRF2* in SNU719 and C666-1 cells after 2, 4, 8, 24, and 48 h of mTZ3-LNP treatment (n = 3 experimental replicates). Data are presented as mean ± SD. Source data are provided as a Source Data file.

Supplementary Figure 7

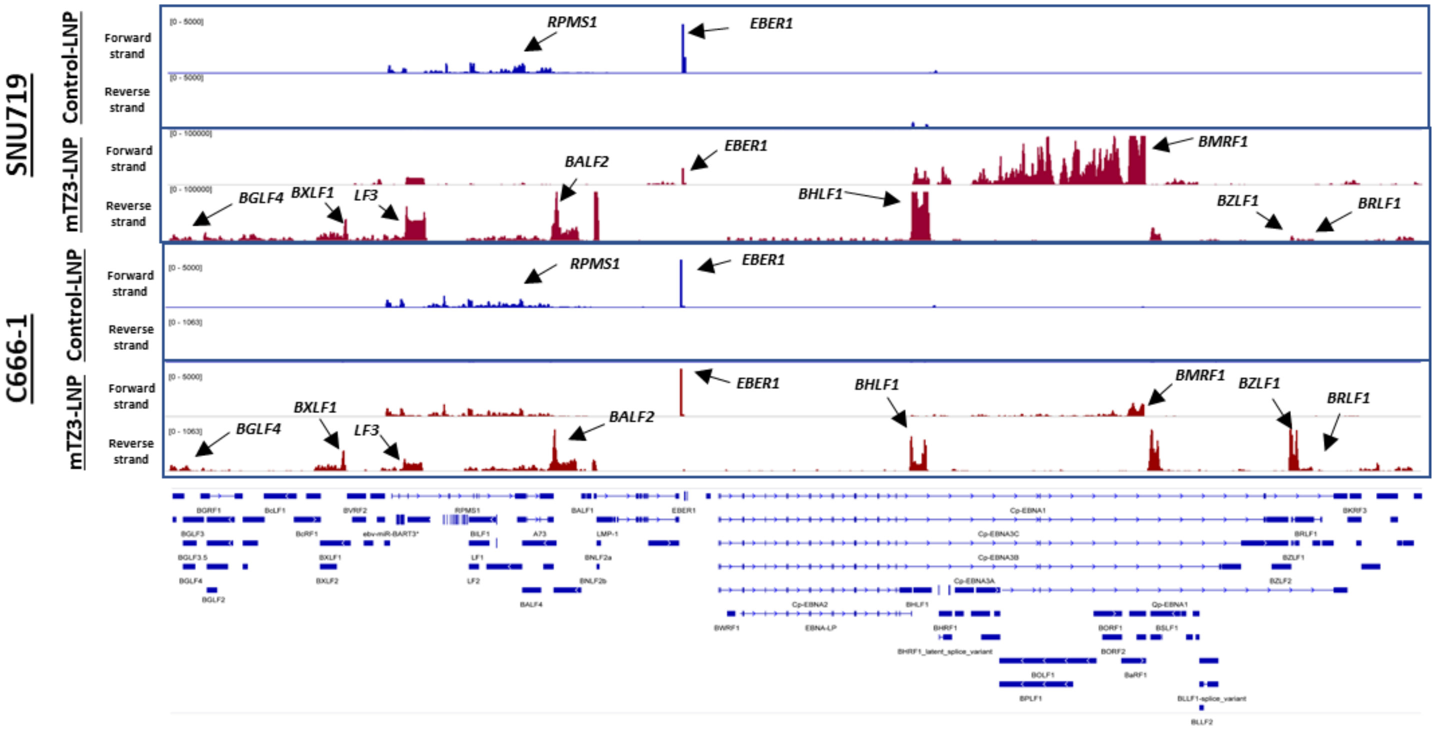

Supplementary Figure 7. Induction of EBV lytic genes in mTZ3-LNP treated EBVaGC and NPC cells. EBV transcriptome profiles illustrating the induction of multiple EBV lytic genes in SNU719 and C666-1 cells after 48 h of mTZ3-LNP treatment (n = 3 experimental replicates). The EBV latent and lytic genes are indicated by the arrows.

Supplementary Figure 8

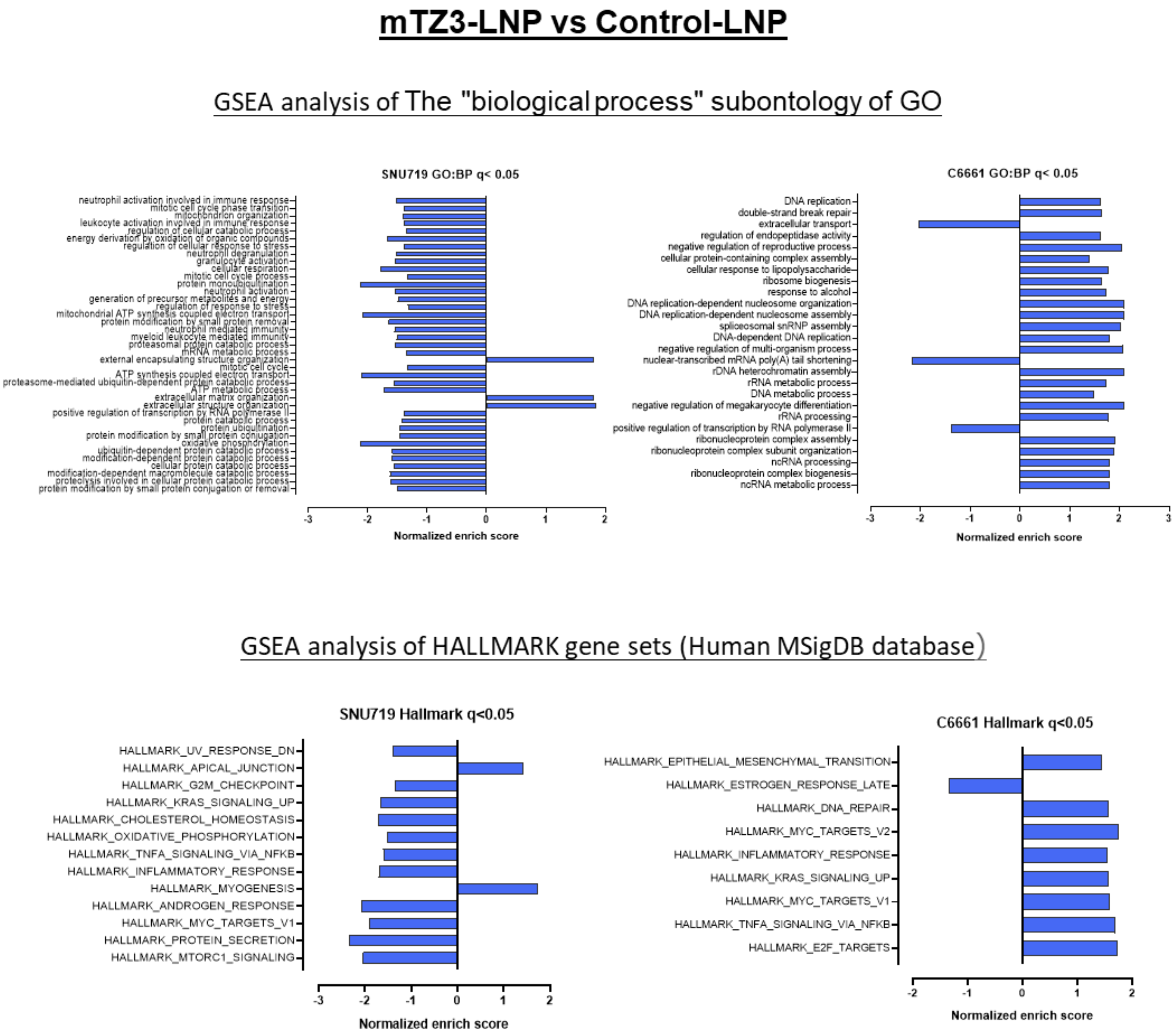

Supplementary Figure 8. GSEA of differentially expressed genes in SNU719 and C666-1 cells after mTZ3-LNP treatment. The differentially expressed gene sets identified in the mTZ3-LNP vs Control-LNP treatment of SNU719 and C666-1 cells were subjected to Gene Set Enrichment Analysis (GSEA) with the “Biological process: subontology of Gene Ontology (GO)” and “HALLMARK” gene sets. A normalized enrichment score <0 indicates that the pathway was downregulated after mTZ3-LNP treatment.

Supplementary Figure 9

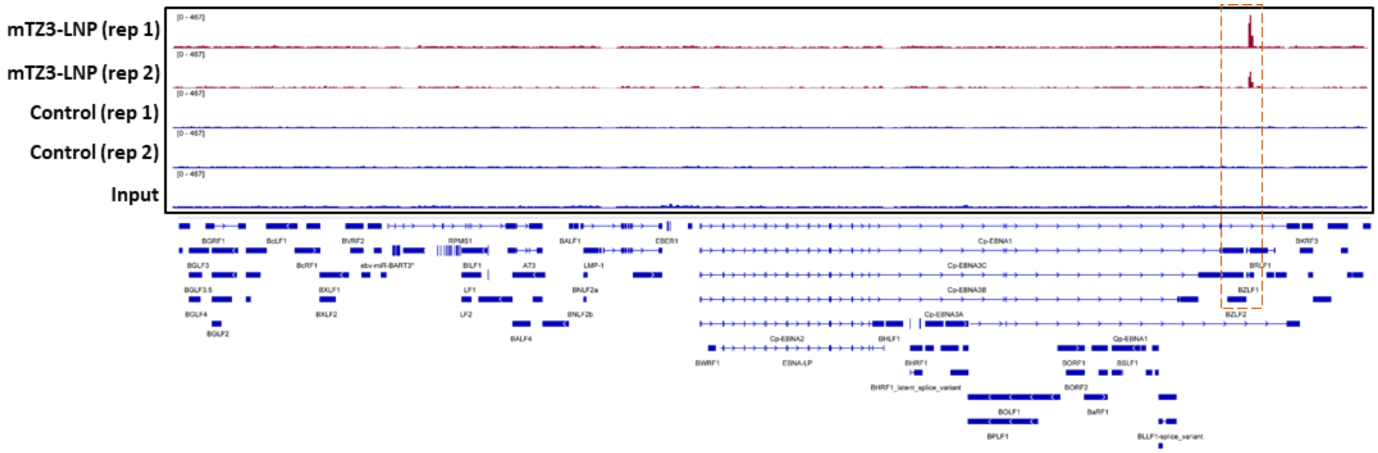

Supplementary Figure 9. Detection of the genomic specificity of the TZ3 TALE transcriptional activator in EBV-positive NPC cells. The genomic specificity of the TZ3 TALE transcriptional activator was evaluated in mTZ3-LNP-treated C666-1 cells using ChIP-sequencing with an anti-FLAG antibody. A single peak was illustrated in the promoter region of BZLF1 in the EBV genome (red open box) from C666-1 cells treated with mTZ3-LNP for 6 h. C666-1 cells treated with control-LNP were included as a control. The experiments were conducted in duplicate.

Supplementary Figure 10

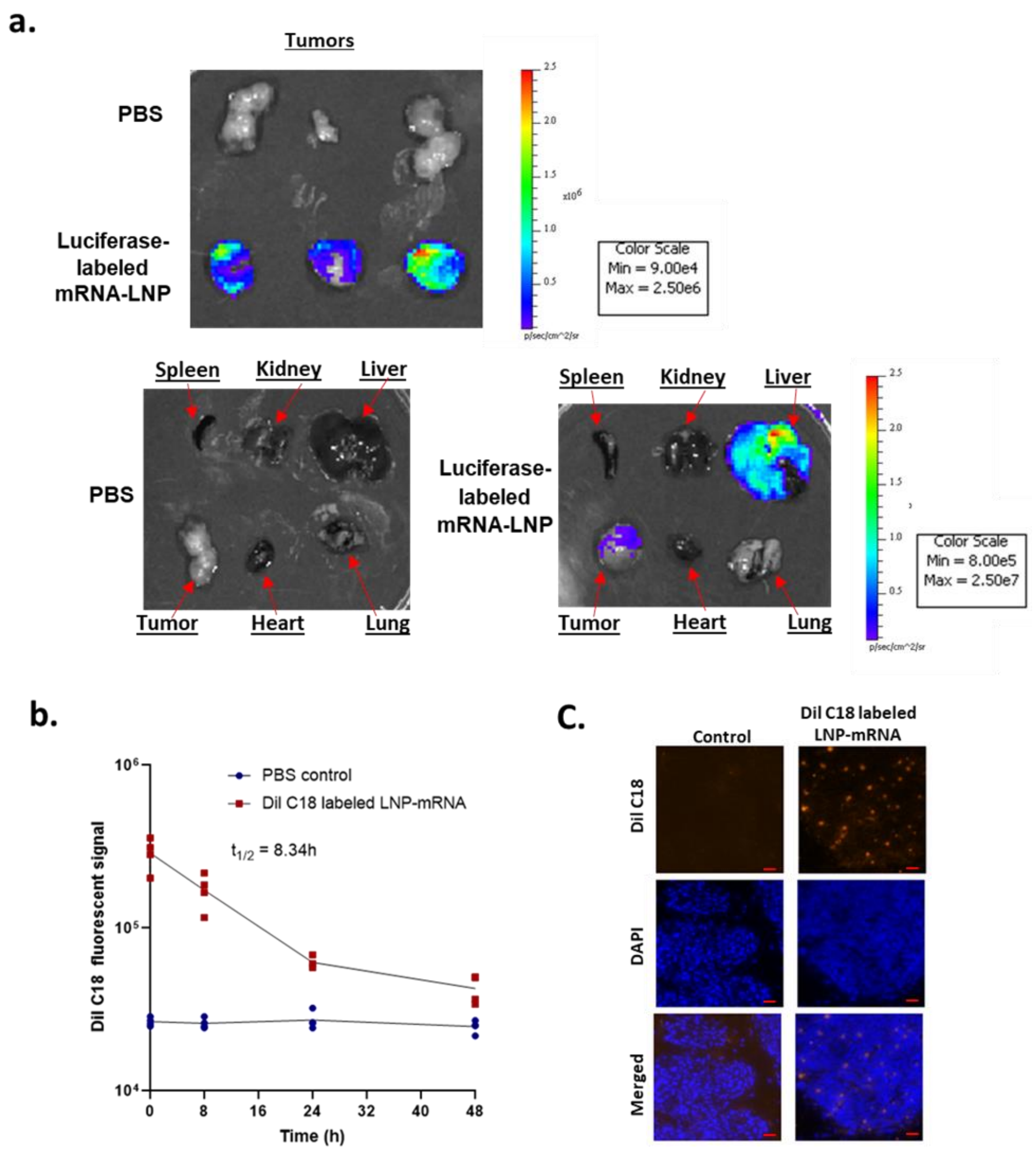

Supplementary Figure 10. Formulated LNP delivers mRNA to *in vivo* tumor xenograft models in NOD-SCID mice. a. The delivery of formulated LNPs encapsulated luciferase-labeled mRNAs into the tumors of C666-1 cell-derived xenograft mouse models. The tumors and normal organs (red arrows) were collected from the mice at 24 h post-intravenous injection of LNP-encapsulated luciferase-labeled mRNA into the mice (n=3 experimental replicates). Luciferase signals were also detected in the tumors, as well as the liver tissues. **b.** The lifetime of the formulated LNP in circulation was measured in the mice intravenously injected with Dil C18-labelled LNP encapsulated *TZ3* mRNA. The Dil C18 fluorescent signals were detected in blood samples collected at 0, 8, 24 and 48 h post injection (n = 3 experimental replicates). Date are presented as mean ± SD.

The half-life of circulating formulated mTZ3-LNP ($t_{1/2}$) was calculated to be 8.34 h. **c.** Representative images show the Dil C18 fluorescent signals in the tumors of SNU719 cell-derived xenograft mouse models. The tumors were collected from the mice at 3 h post-intravenous injection of Dil C18-labelled LNP encapsulated *TZ3* mRNA or PBS control (n = 3 experimental replicates). Scale bar = 20 μm. Source data are provided as a Source Data file.

Supplementary Figure 11

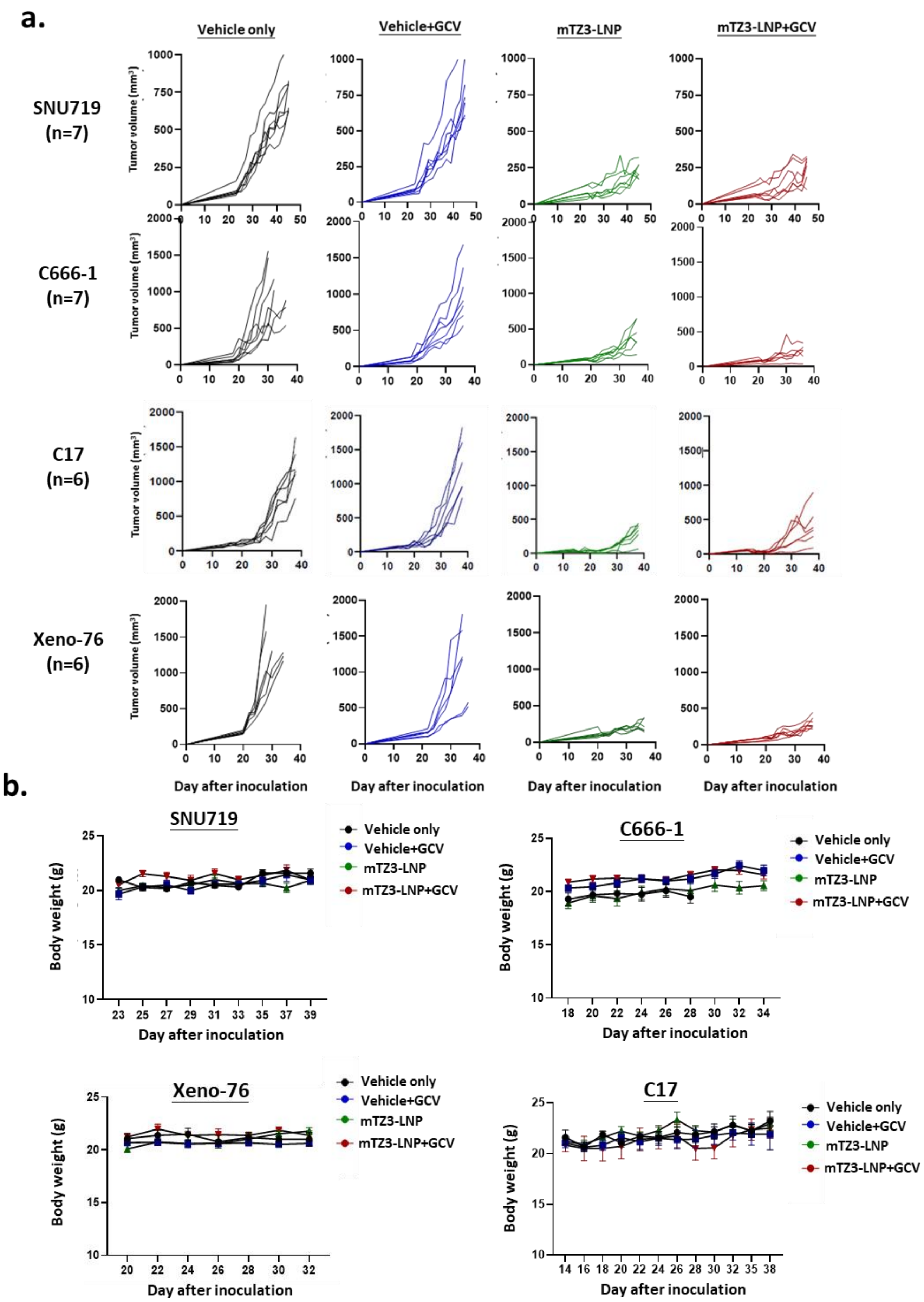

Supplementary Figure 11

Supplementary Figure 11. mRNA-LNP-based lytic induction therapy targeted EBV-positive epithelial cancers. a, The tumor volume and **(b)** body weight were determined in mice implanted with SNU719, C666-1, C17 and Xeno-76 xenografts and treated with vehicle alone, vehicle combined with GCV, mTZ3-LNP alone, or mTZ3-LNP combined with GCV (SNU719 and C666-1: n = 7 mice; C17 and Xeno-76: n = 6 mice). Data are presented as mean ± SEM. Source data are provided as a Source Data file.

Supplementary Figure 12

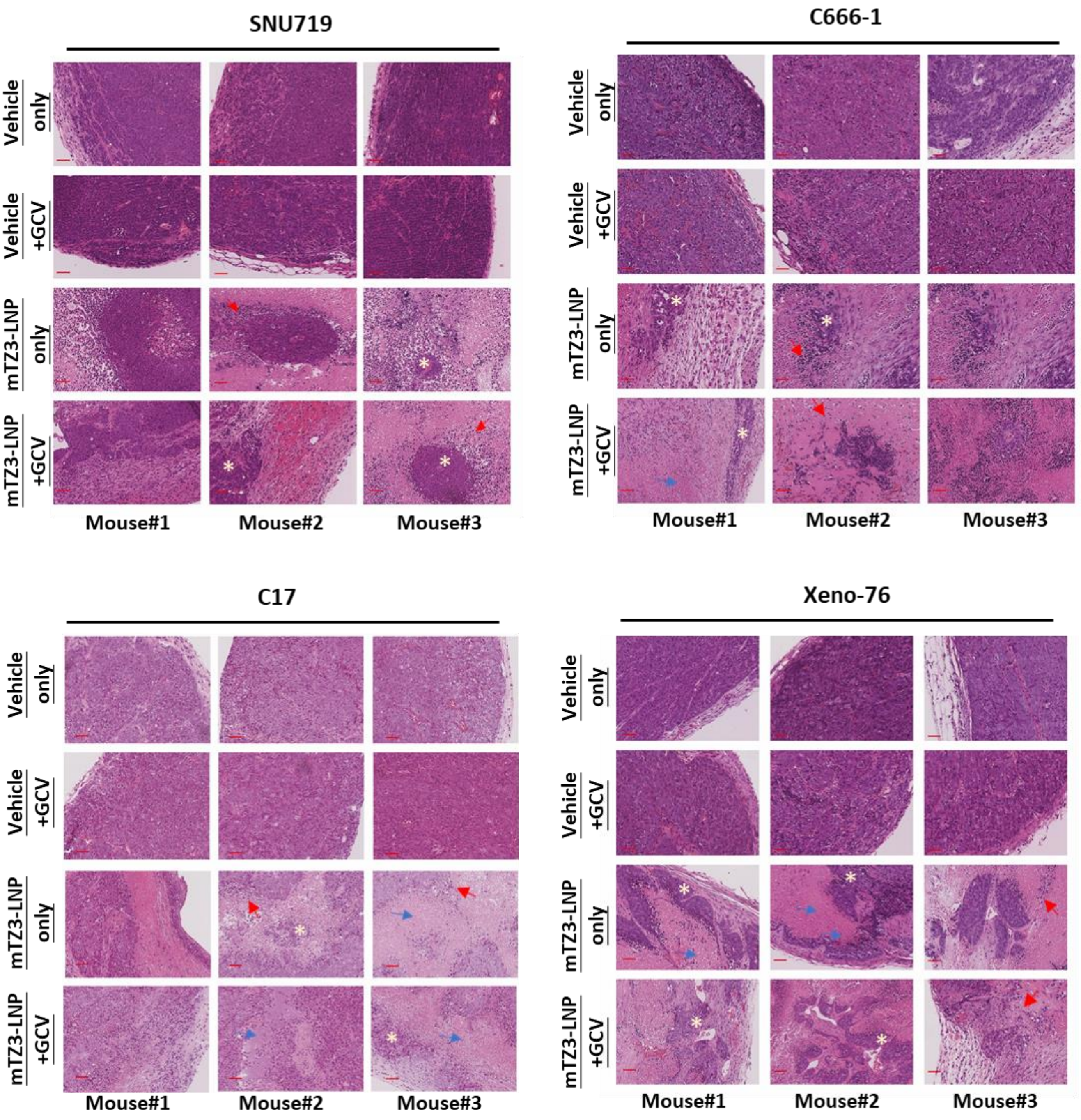

Supplementary Figure 12. The inhibition of EBV-positive epithelial cancers *in vivo* by mTZ3-LNP-based lytic induction treatment. Hematoxylin and eosin staining reveals the EBV-positive tumor cells in representative FFPE sections of residual tumors harvested from mice after treatment. Heavy infiltration of lymphocytes (red arrows) and necrotic lesions (blue arrows) were found in the tumors treated with mTZ3-LNP alone or a combination of mTZ3-LNP and GCV. The residue tumors are indicated by (*). Scale bar = 250 μM. Representative images from each group (SNU719 and C666-1: n = 7 mice/ group; C17 and Xeno-76: n = 6 mice/group) are shown.

Supplementary Figure 13

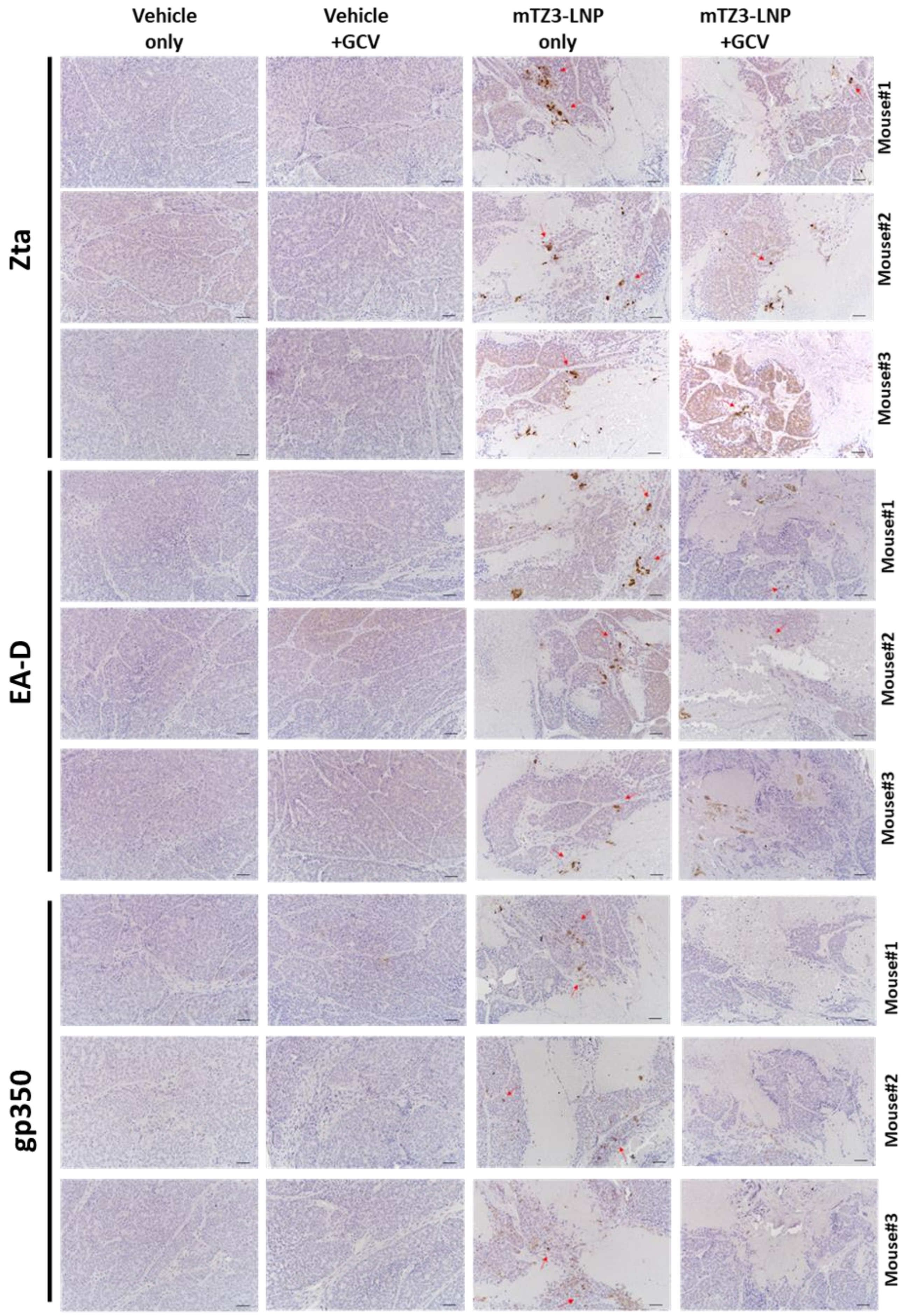

Supplementary Figure 13

Supplementary Figure 13. Expression of Zta, EA-D and gp350 in Xeno-76 tumors after mTZ3-LNP-based lytic induction treatment. Using immunohistochemical staining, the expression of Zta, EA-D and gp350 was detected in the representative tissue sections of Xeno-76 tumor in NOD-SCID mouse models with mTZ3-LNP-based lytic induction treatment. Representative images from n = 6 mice/group are shown. The tumor cells expressing the EBV lytic proteins (Zta, EA-D or gp350) are illustrated by the red arrows. Scale bar = 50 μm.

Supplementary Figure 14

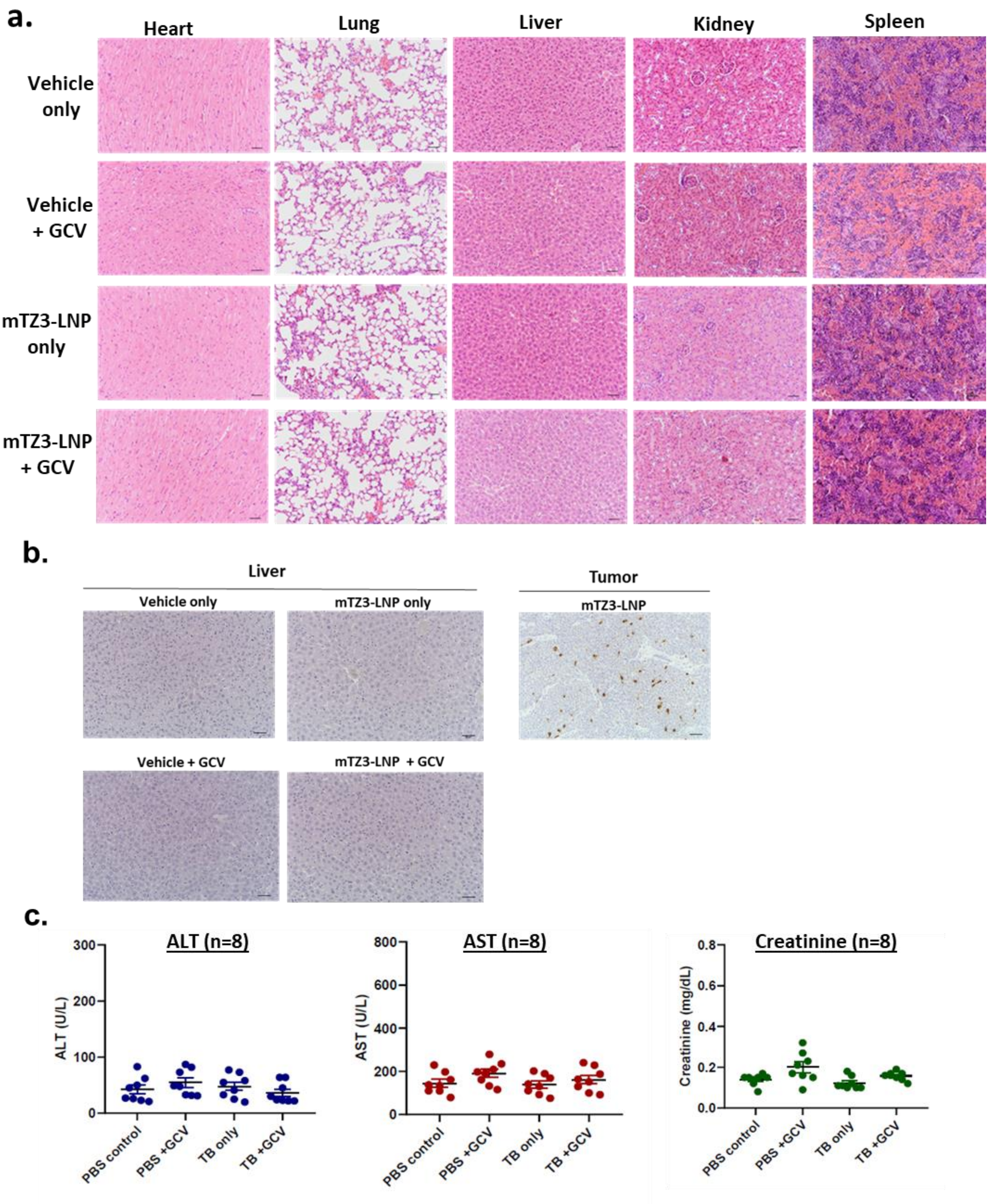

Supplementary Figure 14

Supplementary Figure 14. Effects of mRNA-LNP-based lytic induction therapy on normal organs and the plasma concentrations of ALT, AST and creatinine of NOD-SCID mice. a. Representative images of hematoxylin and eosin stained FFPE sections of organs harvested from mTZ3-LNP and GCV treated mice are shown. No abnormal lesions were observed in mice treated with mTZ3-LNP alone or combined with GCV (n = 8 mice/group). Scale bar = 50 μm. **b,** No Zta expression was detected via immunohistochemical staining of the liver tissues of mice implanted with SNU719 xenografts and treated with mTZ3-LNP alone or combined with GCV. A SUN719 tumor treated with mTZ3 was included as a control (n = 7 mice/group). Representative image of each treatment group is shown. Scale bar = 50 mm. **c.** The plasma concentration of alanine transaminase (ALT), aspartate aminotransferase (AST) and creatinine were determined in mice treated with vehicle alone, vehicle combined with GCV, mTZ3-LNP alone and mTZ3-LNP combined with GCV (n = 8 mice/group). Data are presented as mean ± SEM. The ALT, AST and creatine levels did not differ significantly between the four groups of mice and were similar to the ranges reported for normal untreated mice. The normal ranges for ALT, AST, and creatinine concentrations in the mice are 29-80 U/L, 63-227 U/L, and 0.2-0.5 mg/dL, respectively. Source data are provided as a Source Data file.

Supplementary Figure 15

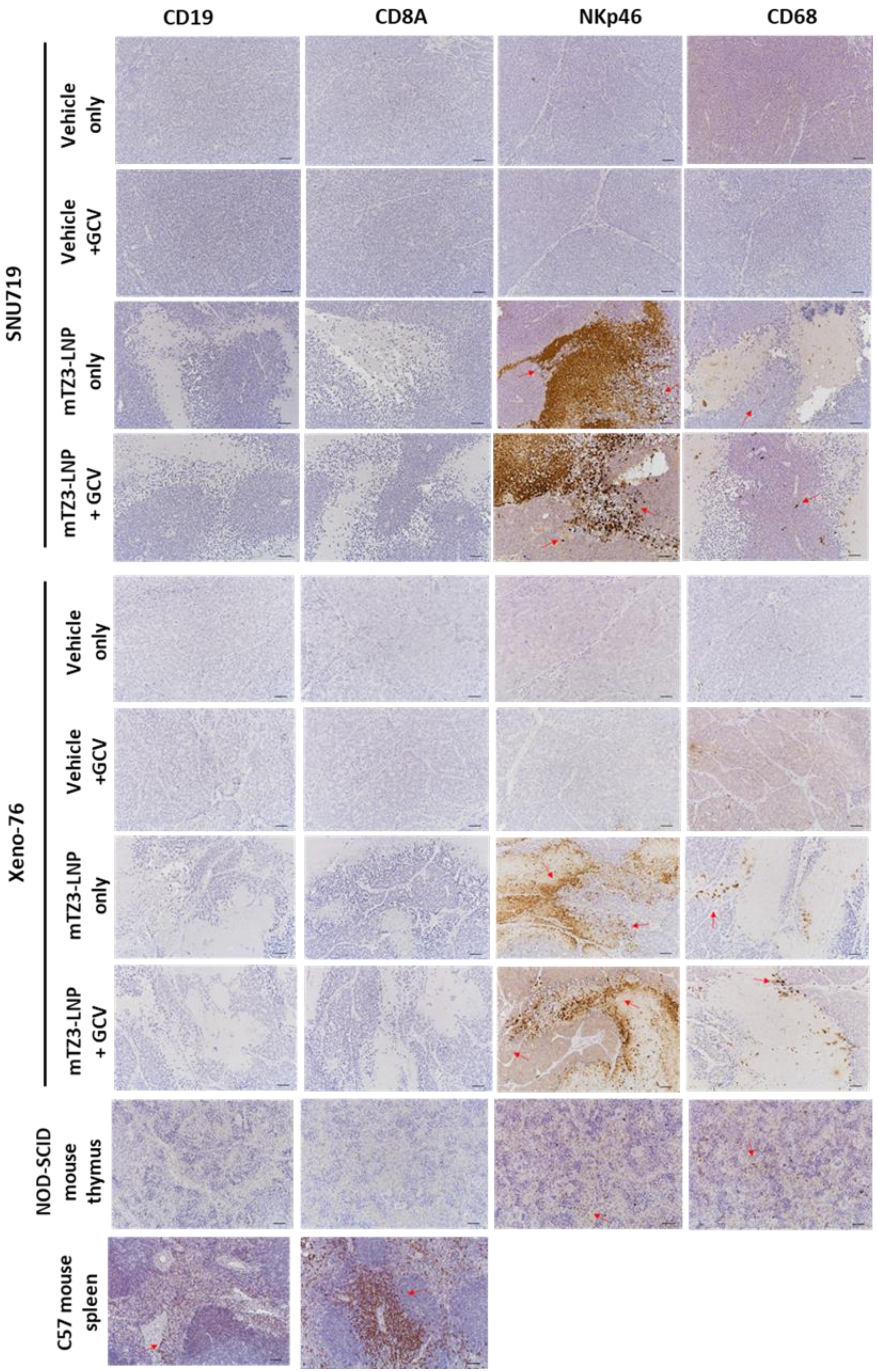

Supplementary Figure 15

Supplementary Figure 15. Characterization of infiltrating lymphocytes in tumors harvested from the mice treated with mTZ3-LNP alone and in combination with GCV. The tumors harvested from SNU719 and Xeno-76 xenograft models treated with mTZ3-LNP alone, combined mTZ3-LNP and GCV, GCV alone and vehicle controls were subjected to immunohistochemical staining of mouse lymphocyte markers, including CD19 (B cells), CD8A (cytotoxic T cells), NKp46 (NK cells) and CD68 (macrophages) (SNU719: n = 7 mice/group; Xeno-76: n = 6 mice/group). Representative images of each treatment group are shown. Spleen tissues from C57 mice were included as a positive control for CD19 and CD8A staining. Neither CD19-positive nor CD8A-positive cells were detected in the tumors or the normal thymus tissues of NOD-SCID mice. Red arrows indicate the positively stained cells. Scale bar = 50 μm.

Supplementary Table 1: Plasmids used in this study

**Casilio Activation System:**	**Description**
pLenti-HA-dCas9-2A-EGFP-2A-Blast	lentiviral vector constitutively expressing HA-dCas9-2A-EGFP
pLX-3xFLAG-4xNLS-PUFa-2xNLS-p65HSF1-IRES-Hygro	lentiviral vector constitutively expressing 3xFLAG-PUFa-p65HSF1 transactivator
**sgRNAs:**	**sgRNA-target sequence (added 5' g in lower case) Target sequence PAM**
pLentiGuide-sgBZLF1-1-5xPBSa (sgRNA1)	GCAAAGATAGCAAAGGTGGC **CGG**
pLentiGuide-sgBZLF1-2-5xPBSa (sgRNA2)	gCAGCCTCCTCTGTGATGTCA **TGG**
pLentiGuide-sgBZLF1-3-5xPBSa (sgRNA3)	GAAACTATGCATGAGCCAC **AGG**
pLentiGuide-sgBZLF1-4-5xPBSa (sgRNA4)	gCAGAAGTGTCTAAAATAAGC **TGG**
**Inducible 3xFLAG-PUFa-p65HSF piggyBac plasmid:**	**Description**
PB-Hygro_2a_rtTA3-3xFLAG-4xNLS-PUFa-2xNLS-p65HSF1	piggyBac transposon vector expressing rtTA3 and Tet-On 3xFLAG-PUFa-p65HSF transactivator
**TALE plasmids**	**Target sequence**
pcDNA3.1-3xFLAG-4xNLS_TALE-BZLP1-1_2xNLS_p65HSF1 (TZ1)	TAGCAAAGGTGGCCGG
pcDNA3.1-3xFLAG-4xNLS_TALE-BZLP1-2_2xNLS_p65HSF1 (TZ2)	TCCTCTGTGATGTCATGG
pcDNA3.1-3xFLAG-4xNLS_TALE-BZLP1-3_2xNLS_p65HSF1 (TZ3)	TATGCATGAGCCACAGG
pcDNA3.1-3xFLAG-4xNLS_TALE-BZLP1-4_2xNLS_p65HSF1 (TZ4)	TGTCTAAAATAAGCTGG

Supplementary table 2. sgRNA- and TALE-binding sequences in the *BZLF1* promoter

**sgRNA-binding sequences**	
**sg1**	**5' - ggcca ccttt gctat cttt - 3'**
**sg2**	**5' - tgaca tcaca gagga ggc - 3'**
**sg3**	**5' - aaact atgca tgagc cac - 3'**
**sg4**	**5'- gctta tttta gacac ttc - 3'**
**TALE-binding sequences**	
**TZ1**	**tagcaaaggtggccgg**
**TZ2**	**tcctctgtgatgtcatgg**
**TZ3**	**tatgcatgagccacagg**
**TZ4**	**tgtctaaaataagctgg**

Supplementary table 3. Amino acid sequences of the TALEs and the RNA sequence of the TZ3 mRNA.

TZ1<br>amino<br>acid<br>sequence	MDYKDHDGDYKDHDIDYKDDDDKIDGGGGSDPKKKRKVDPKKKRKVDPKKKRKVGSTGSRNDGGGGSGGGGSGGG<br>GSGRAVDLRTLGYSQQQQEKIKPKVRSTVAQHHEALVGHGFTHAHIVALSQHPAALGTVAVTYQHIITALPEATHEDIV<br>GVGKQWSGARALEALLTDAGELRGPPLQLDTGQLVKIAKRGGVTAMEAVHASRNALTGAPLNLTPDQVVAIASNIGGK<br>QALETVQRLLPVLCQDHGLTPDQVVAIASNNGGKQALETVQRLLPVLCQDHGLTPDQVVAIASHDGGKQALETVQRLL<br>PVLCQDHGLTPDQVVAIASNIGGKQALETVQRLLPVLCQDHGLTPDQVVAIASNIGGKQALETVQRLLPVLCQDHGLTP<br>DQVVAIASNIGGKQALETVQRLLPVLCQDHGLTPDQVVAIASNNGGKQALETVQRLLPVLCQDHGLTPDQVVAIASNN<br>GGKQALETVQRLLPVLCQDHGLTPDQVVAIASNGGGKQALETVQRLLPVLCQDHGLTPDQVVAIASNNGGKQALETV<br>QRLLPVLCQDHGLTPDQVVAIASNNGGKQALETVQRLLPVLCQDHGLTPDQVVAIASHDGGKQALETVQRLLPVLCQD<br>HGLTPDQVVAIASHDGGKQALETVQRLLPVLCQDHGLTPDQVVAIASNNGGKQALETVQRLLPVLCQDHGLTPDQVV<br>AIASNNGGKQALESIVAQLSRPDPALAALTNDHLVALACLGGRPAMDAVKKGLPHAPELIRRVNRRIGERTSHRVARDP<br>KKKRKVDPKKKRKVGGRGGGGSGGGGSGGGGSGPAGGGGSGGGGSGGGGSGPKKKRKVAAAGSPSGQISNQALAL<br>APSSAPVLAQTMVPSSAMVPLAQPPAPAPVLTPGPPQSLSAPVPKSTQAGEGTLSEALLHLQFDADEDLGALLGNSTDP<br>GVFTDLASVDNSEFQQLLNQGVSMSHSTAEPMLMEYPEAITRLVTGSQRPPDPAPTPLGTSGLPNGLSGDEDFSSIAD<br>MDFSALLSQISSSGQGGGGSGFSVDTSALLDLFSPSVTVPDMSLPDLDSSLASIQELLSPQEPPRPPEAENSSPDSGKQLV<br>HYTAQPLFLLDPGSVDTGSNDLPVLFELGEGSYFSEGDGFAEDPTISLLTGSEPPKAKDPTVSID*
TZ2<br>amino<br>acid<br>sequence	MDYKDHDGDYKDHDIDYKDDDDKIDGGGGSDPKKKRKVDPKKKRKVDPKKKRKVGSTGSRNDGGGGSGGGGSGGG<br>GSGRAVDLRTLGYSQQQQEKIKPKVRSTVAQHHEALVGHGFTHAHIVALSQHPAALGTVAVTYQHIITALPEATHEDIV<br>GVGKQWSGARALEALLTDAGELRGPPLQLDTGQLVKIAKRGGVTAMEAVHASRNALTGAPLNLTPDQVVAIASNIGGK<br>QALETVQRLLPVLCQDHGLTPDQVVAIASNNGGKQALETVQRLLPVLCQDHGLTPDQVVAIASHDGGKQALETVQRLL<br>PVLCQDHGLTPDQVVAIASNIGGKQALETVQRLLPVLCQDHGLTPDQVVAIASNIGGKQALETVQRLLPVLCQDHGLTP<br>DQVVAIASNIGGKQALETVQRLLPVLCQDHGLTPDQVVAIASNNGGKQALETVQRLLPVLCQDHGLTPDQVVAIASNN<br>GGKQALETVQRLLPVLCQDHGLTPDQVVAIASNGGGKQALETVQRLLPVLCQDHGLTPDQVVAIASNNGGKQALETV<br>QRLLPVLCQDHGLTPDQVVAIASNNGGKQALETVQRLLPVLCQDHGLTPDQVVAIASHDGGKQALETVQRLLPVLCQD<br>HGLTPDQVVAIASHDGGKQALETVQRLLPVLCQDHGLTPDQVVAIASNNGGKQALETVQRLLPVLCQDHGLTPDQVV<br>AIASNNGGKQALESIVAQLSRPDPALAALTNDHLVALACLGGRPAMDAVKKGLPHAPELIRRVNRRIGERTSHRVARDP<br>KKKRKVDPKKKRKVGGRGGGGSGGGGSGGGGSGPAGGGGSGGGGSGGGGSGPKKKRKVAAAGSPSGQISNQALAL<br>APSSAPVLAQTMVPSSMVPLAQPPAPAPVLTPGPPQSLSAPVPKSTQAGEGTLSEALLHLQFDADEDLGALLGNSTDPG<br>VFTDLASVDNSEFQQLLNQGVSMSHSTAEPMLMEYPEAITRLVTGSQRPPDPAPTPLGTSGLPNGLSGDEDFSSIADM<br>DFSALLSQISSSGQGGGGSGFSVDTSALLDLFSPSVTVPDMSLPDLDSSLASIQELLSPQEPPRPPEAENSSPDSGKQLVHY<br>TAQPLFLLDPGSVDTGSNDLPVLFELGEGSYFSEGDGFAEDPTISLLTGSEPPKAKDPTVSID*
TZ3<br>amino<br>acid<br>sequence	MDYKDHDGDYKDHDIDYKDDDDKIDGGGGSDPKKKRKVDPKKKRKVDPKKKRKVGSTGSRNDGGGGSGGGGSGGG<br>GSGRAVDLRTLGYSQQQQEKIKPKVRSTVAQHHEALVGHGFTHAHIVALSQHPAALGTVAVTYQHIITALPEATHEDIV<br>GVGKQWSGARALEALLTDAGELRGPPLQLDTGQLVKIAKRGGVTAMEAVHASRNALTGAPLNLTPDQVVAIASNIGGK<br>QALETVQRLLPVLCQDHGLTPDQVVAIASNGGGKQALETVQRLLPVLCQDHGLTPDQVVAIASNNGGKQALETVQRLL<br>PVLCQDHGLTPDQVVAIASHDGGKQALETVQRLLPVLCQDHGLTPDQVVAIASNIGGKQALETVQRLLPVLCQDHGLT<br>PDQVVAIASNGGGKQALETVQRLLPVLCQDHGLTPDQVVAIASNNGGKQALETVQRLLPVLCQDHGLTPDQVVAIASN<br>IGGKQALETVQRLLPVLCQDHGLTPDQVVAIASNNGGKQALETVQRLLPVLCQDHGLTPDQVVAIASHDGGKQALETV<br>QRLLPVLCQDHGLTPDQVVAIASHDGGKQALETVQRLLPVLCQDHGLTPDQVVAIASNIGGKQALETVQRLLPVLCQD<br>HGLTPDQVVAIASHDGGKQALETVQRLLPVLCQDHGLTPDQVVAIASNIGGKQALETVQRLLPVLCQDHGLTPDQVVAI<br>ASNNGGKQALETVQRLLPVLCQDHGLTPDQVVAIASNNGGKQALESIVAQLSRPDPALAALTNDHLVALACLGGRPAM<br>DAVKKGLPHAPELIRRVNRRIGERTSHRVARDPKKKRKVDPKKKRKVGGRGGGGSGGGGSGGGGSGPAGGGGSGGG<br>GSGGGGSGPKKKRKVAAAGSPSGQISNQALALAPSSAPVLAQTMVPSSAMVPLAQPPAPAPVLTPGPPQSLSAPVPKS<br>TQAGEGTLSEALLHLQFDADEDLGALLGNSTDPGVFTDLASVDNSEFQQLLNQGVSMSHSTAEPMLMEYPEAITRLVT<br>GSQRPPDPAPTPLGTSGLPNGLSGDEDFSSIADMDFSALLSQISSSGQGGGGSGFSVDTSALLDLFSPSVTVPDMSLPDL<br>DSSLASIQELLSPQEPPRPPEAENSSPDSGKQLVHYTAQPLFLLDPGSVDTGSNDLPVLFELGEGSYFSEGDGFAEDPT<br>ISLLTGSEPPKAKDPTVSID*

TZ4 amino acid sequence	MDYKDHDGDYKDHDIDYKDDDDKIDGGGGSDPKKKRKVDPKKKRKVDPKKKRKVGSTGSRNDGGGGSGGGGSGGG<br>GSGRAVDLRTLGYSQQQQEKIKPKVRSTVAQHHEALVGHGFTHAHIVALSQHPAALGTVAVTYQHIITALPEATHEDIV<br>GVGKQWSGARALEALLTDAGELRGPPLQLDTGQLVKIAKRGGVTAMEAVHASRNALTGAPLNLTPDQVVAIASNNGG<br>KQALETVQRLLPVLCQDHGLTPDQVVAIASNGGGKQALETVQRLLPVLCQDHGLTPDQVVAIASHDGGKQALETVQRL<br>LPVLCQDHGLTPDQVVAIASNGGGKQALETVQRLLPVLCQDHGLTPDQVVAIASNIGGKQALETVQRLLPVLCQDHGLT<br>PDQVVAIASNIGGKQALETVQRLLPVLCQDHGLTPDQVVAIASNIGGKQALETVQRLLPVLCQDHGLTPDQVVAIASNI<br>GGKQALETVQRLLPVLCQDHGLTPDQVVAIASNGGGKQALETVQRLLPVLCQDHGLTPDQVVAIASNIGGKQALETVQ<br>RLLPVLCQDHGLTPDQVVAIASNIGGKQALETVQRLLPVLCQDHGLTPDQVVAIASNNGGKQALETVQRLLPVLCQDH<br>GLTPDQVVAIASHDGGKQALETVQRLLPVLCQDHGLTPDQVVAIASNGGGKQALETVQRLLPVLCQDHGLTPDQVVAI<br>ASNNGGKQALETVQRLLPVLCQDHGLTPDQVVAIASNNGGKQALESIVAQLSRPDPALAALTNDHLVALACLGGRPAM<br>DAVKKGLPHAPELIRRVNRRIGERTSHRVARDPKKKRKVDPKKKRKVGGRGGGGSGGGGSGGGGSGPAGGGGSGGG<br>GSGGGGSGPKKKRKVAAAGSPSGQISNQALALAPSSAPVLAQTMVPSSAMVPLAQPPAPAPVLTPGPPQSLSAPVPKS<br>TQAGEGTLSEALLHLQFDADEDLGALLGNSTDPGVFTDLASVDNSEFQQLLNQGVSMSHSTAEPMLMEYPEAITRLVT<br>GSQRPPDPAPTPLGTSGLPNGLSGDEDFSSIADMDFSALLSQISSSGQGGGGSGFSVDTSALLDLFSPSVTVPDMSLPDL<br>DSSLASIQELLSPQEPPRPPEAENSSPDSGKQLVHYTAQPLFLLDPGSVDTGSNDLPVLFELGEGSYFSEGDGFAEDPTI<br>SLLTGSEPPKAKDPTVSID*
TZ3 mRNA	AUGGACUACAAGGAUCACGACGGUGACUAUAAGGAUCAUGACAUCGACUAUAAGGACGAUGACGAUAAGAU<br>CGAUGGCGGAGGCGGAUCUGAUCCAAAAAAGAAGAGAAAGGUAGAUCCAAAAAAGAAGAGAAAGGUAGAUC<br>CAAAAAAGAAGAGAAAGGUAGGAUCUACCGGAUCUAGAAACGAUGGUGGUGGUGGAAGCGGGGGUGGGGG<br>CAGCGGUGGAGGGGGAAGCGGGCGCGCCGUGGAUCUACGCACGCUCGGCUACAGUCAGCAGCAGCAAGAGAA<br>GAUCAAACCGAAGGUGCGUUCGACAGUGGCGCAGCACCACGAGGCACUGGUGGGCCAUGGGUUUACACACGC<br>GCACAUCGUUGCGCUCAGCCAACACCCGGCAGCGUUAGGGACCGUCGCUGUCACGUAUCAGCACAUAAUCAC<br>GGCGUUGCCAGAGGCGACACACGAAGACAUCGUUGGCGUCGGCAAACAGUGGUCCGGCGCACGCGCCCUGGA<br>GGCCUUGCUCACGGAUGCGGGGGAGUUGAGAGGUCCGCCGUUACAGUUGGACACAGGCCAACUUGUGAAGA<br>UUGCAAAACGUGGCGGCGUGACCGCAAUGGAGGCAGUGCAUGCAUCGCGCAAUGCACUGACGGGUGCCCCC<br>UGAACCUGACCCCGGACCAAGUGGUGGCUAUCGCCAGCAACAUUGGCGGCAAGCAAGCGCUCGAAACGGUGC<br>AGCGGCUGUUGCCGGUGCUGUGCCAGGACCAUGGCCUGACCCCGGACCAAGUGGUGGCUAUCGCCAGCAACG<br>GUGGCGGCAAGCAAGCGCUCGAAACGGUGCAGCGGCUGUUGCCGGUGCUGUGCCAGGACCAUGGCCUGACCC<br>CGGACCAAGUGGUGGCUAUCGCCAGCAAUCACGGCGGCAAGCAAGCGCUCGAAACGGUGCAGCGGCUGUUGC<br>CGGUGCUGUGCCAGGACCAUGGCCUGACUCCGGACCAAGUGGUGGCUAUCGCCAGCCACGAUGGCGGCAAGC<br>AAGCGCUCGAAACGGUGCAGCGGCUGUUGCCGGUGCUGUGCCAGGACCAUGGCCUGACCCCGGACCAAGUGG<br>UGGCUAUCGCCAGCAACAUUGGCGGCAAGCAAGCGCUCGAAACGGUGCAGCGGCUGUUGCCGGUGCUGUGC<br>CAGGACCAUGGCCUGACCCCGGACCAAGUGGUGGCUAUCGCCAGCAACGGUGGCGGCAAGCAAGCGCUCGAA<br>ACGGUGCAGCGGCUGUUGCCGGUGCUGUGCCAGGACCAUGGCCUGACCCCGGACCAAGUGGUGGCUAUCGC<br>CAGCAAUCACGGCGGCAAGCAAGCGCUCGAAACGGUGCAGCGGCUGUUGCCGGUGCUGUGCCAGGACCAUGG<br>CCUGACCCCGGACCAAGUGGUGGCUAUCGCCAGCAACAUUGGCGGCAAGCAAGCGCUCGAAACGGUGCAGCG<br>GCUGUUGCCGGUGCUGUGCCAGGACCAUGGCCUGACCCCGGACCAAGUGGUGGCUAUCGCCAGCAAUCACGG<br>CGGCAAGCAAGCGCUCGAAACGGUGCAGCGGCUGUUGCCGGUGCUGUGCCAGGACCAUGGCCUGACUCCGGA<br>CCAAGUGGUGGCUAUCGCCAGCCACGAUGGCGGCAAGCAAGCGCUCGAAACGGUGCAGCGGCUGUUGCCGGU<br>GCUGUGCCAGGACCAUGGCCUGACCCCGGACCAAGUGGUGGCUAUCGCCAGCCACGAUGGCGGCAAGCAAGC<br>GCUCGAAACGGUGCAGCGGCUGUUGCCGGUGCUGUGCCAGGACCAUGGCCUGACCCCGGACCAAGUGGUGG<br>CUAUCGCCAGCAACAUUGGCGGCAAGCAAGCGCUCGAAACGGUGCAGCGGCUGUUGCCGGUGCUGUGCCAGG<br>ACCAUGGCCUGACUCCGGACCAAGUGGUGGCUAUCGCCAGCCACGAUGGCGGCAAGCAAGCGCUCGAAACGG<br>UGCAGCGGCUGUUGCCGGUGCUGUGCCAGGACCAUGGCCUGACCCCGGACCAAGUGGUGGCUAUCGCCAGCA<br>ACAUUGGCGGCAAGCAAGCGCUCGAAACGGUGCAGCGGCUGUUGCCGGUGCUGUGCCAGGACCAUGGCCUG<br>ACCCCGGACCAAGUGGUGGCUAUCGCCAGCAAUCACGGCGGCAAGCAAGCGCUCGAAACGGUGCAGCGGCUG<br>UUGCCGGUGCUGUGCCAGGACCAUGGCCUGACCCCGGACCAAGUGGUGGCUAUCGCCAGCAAUCACGGCGGC<br>AAGCAAGCGCUCGAAAGCAUUGUGGCCCAGCUGAGCCGGCCUGAUCCGGCGUUGGCCGCGUUGACCAACGAC<br>CACCUCGUCGCCUUGGCCUGCCUCGGCGGACGUCCUGCCAUGGAUGCAGUGAAAAAGGGAUUGCCGCACGCG

	CCGGAAUUGAUCAGAAGAGUCAAUCGCCGUAUUGGCGAACGCACGUCCCAUCGCGUUGCCAGGGACCCAAAG<br>AAGAAGCGCAAAGUGGAUCCUAAAAAGAAAAGAAAGGUAGGCGGCCGCGGGGGAGGCGGUUCCGGUGGCGG<br>CGGAAGCGGAGGUGGAGGAUCAGGGCCGGCCGGAGGAGGUGGAAGCGGAGGAGGAGGAAGCGGAGGAGGA<br>GGUAGCGGACCUAAGAAAAAGAGGAAGGUGGCGGCCGCUGGAUCCCCUUCAGGGCAGAUCAGCAACCAGGCC<br>CUGGCUCUGGCCCCUAGCUCCGCUCCAGUGCUGGCCCAGACUAUGGUGCCCUCUAGUGCUAUGGUGCCUCUG<br>GCCCAGCCACCUGCUCCAGCCCCUGUGCUGACCCCAGGACCACCCCAGUCACUGAGCGCUCCAGUGCCCAAGUC<br>UACACAGGCCGGCGAGGGGACUCUGAGUGAAGCUCUGCUGCACCUGCAGUUCGACGCUGAUGAGGACCUGG<br>GAGCUCUGCUGGGGAACAGCACCGAUCCCGGAGUGUUCACAGAUCUGGCCUCCGUGGACAACUCUGAGUUU<br>CAGCAGCUGCUGAAUCAGGGCGUGUCCAUGUCUCAUAGUACAGCCGAACCAAUGCUGAUGGAGUACCCCGAA<br>GCCAUUACCCGGCUGGUGACCGGCAGCCAGCGGCCCCCCGACCCCGCUCCAACUCCCCUGGGAACCAGCGGCCU<br>GCCUAAUGGGCUGUCCGGAGAUGAAGACUUCUCAAGCAUCGCUGAUAUGGACUUUAGUGCCCUGCUGUCAC<br>AGAUUUCCUCUAGUGGGCAGGGAGGAGGUGGAAGCGGCUUCAGCGUGGACACCAGUGCCCUGCUGGACCUG<br>UUCAGCCCCUCGGUGACCGUGCCCGACAUGAGCCUGCCUGACCUUGACAGCAGCCUGGCCAGUAUCCAAGAG<br>CUCCUGUCUCCCCAGGAGCCCCCCAGGCCUCCCGAGGCAGAGAACAGCAGCCCGGAUUCAGGGAAGCAGCUGG<br>UGCACUACACAGCGCAGCCGCUGUUCCUGCUGGACCCCGGCUCCGUGGACACCGGGAGCAACGACCUGCCGG<br>UGCUGUUUGAGCUGGGAGAGGGCUCCUACUUCUCCGAAGGGGACGGCUUCGCCGAGGACCCCACCAUCUCCC<br>UGCUGACAGGCUCGGAGCCUCCCAAAGCCAAGGACCCCACUGUCUCCAUCGAUUGA

Supplementary table 4. Primer sequences for quantitative RT-PCR

**Genes**	**Primers for qRT-PCR**
BRLF1	For 5'-GCATGGGCGGGACAATCGCAATATAA -3'
	Rev 5'-CCAGCCAGATGTTCAGGAACCAAA -3'
BZLF1	For 5'- TACAAGAATCGGGTGGCTTC -3'
	Rev 5'- GCACATCTGCTTCAACAGGA -3
BMRF1	For 5' - CACTGCGGTGGAGGTAGAG -3'
	Rev 5'- GGTGGTGTGCCATACAAGG -3'
BLRF2	For 5' - ACTGAAGCCCAGGACCAGTTCTA -3'
	Rev 5' - TAAGACAAGCGTCAGAAGTGCCCA -3'
BGLF4	For 5'- GCTGACTCCACCCACAAAAT -3'
	Rev 5'- GAGGTCAGGCCCATGTCTAA -3'
GAPDH	For 5'- GAAGGTGAAGGTCGGAGTCA -3'
	Rev 5' – TGACAAGCTTCCGGTTCTC -3'
