# Supplementary material for: Synthetic BZLF1-targeted transcriptional activator for efficient lytic induction therapy against EBV-associated epithelial cancers
Source: Nat Commun. 2024 May 3;15:3729. doi: 10.1038/s41467-024-48031-8 (PMC11068728; doi:10.1038/s41467-024-48031-8)
Supplement: Supplementary file 3 — Description of Additional Supplementary Files [file 41467_2024_48031_MOESM3_ESM.pdf]

Description of Additional Supplementary Files

Supplementary Data Legends:

Supplementary Data 1. Differentially expressed genes induced by mTZ3-LNP treatment in SNU719 cells.

Supplementary Data 2. Differentially expressed genes induced by mTZ3-LNP treatment in C666-1 cells
