# Supplementary material for: Synthetic BZLF1-targeted transcriptional activator for efficient lytic induction therapy against EBV-associated epithelial cancers
Source: Nat Commun. 2024 May 3;15:3729. doi: 10.1038/s41467-024-48031-8 (PMC11068728; doi:10.1038/s41467-024-48031-8)
Supplement: Supplementary file 6 — Reporting Summary [file 41467_2024_48031_MOESM6_ESM.pdf]

nature portfolio

Corresponding author(s): Kwok-Wai Lo and Albert Wu Cheng

Last updated by author(s): Mar 15, 2024

Reporting Summary

Nature Portfolio wishes to improve the reproducibility of the work that we publish. This form provides structure for consistency and transparency in reporting. For further information on Nature Portfolio policies, see our Editorial Policies and the Editorial Policy Checklist.

Statistics

For all statistical analyses, confirm that the following items are present in the figure legend, table legend, main text, or Methods section.

n/a	Confirmed	
☐	☒	The exact sample size (*n*) for each experimental group/condition, given as a discrete number and unit of measurement
☐	☒	A statement on whether measurements were taken from distinct samples or whether the same sample was measured repeatedly
☐	☒	The statistical test(s) used AND whether they are one- or two-sided<br>*Only common tests should be described solely by name; describe more complex techniques in the Methods section.*
☒	☐	A description of all covariates tested
☐	☒	A description of any assumptions or corrections, such as tests of normality and adjustment for multiple comparisons
☐	☒	A full description of the statistical parameters including central tendency (e.g. means) or other basic estimates (e.g. regression coefficient) AND variation (e.g. standard deviation) or associated estimates of uncertainty (e.g. confidence intervals)
☐	☒	For null hypothesis testing, the test statistic (e.g. *F*, *t*, *r*) with confidence intervals, effect sizes, degrees of freedom and *P* value noted<br>*Give P values as exact values whenever suitable.*
☒	☐	For Bayesian analysis, information on the choice of priors and Markov chain Monte Carlo settings
☒	☐	For hierarchical and complex designs, identification of the appropriate level for tests and full reporting of outcomes
☒	☐	Estimates of effect sizes (e.g. Cohen's *d*, Pearson's *r*), indicating how they were calculated

Our web collection on statistics for biologists contains articles on many of the points above.

Software and code

Policy information about availability of computer code

Data collection	Flow cytometry data was collected using BD LSR Fortessa flow cytometer with FACSDiva software v9. qPCR data was collected using Roche LightCycler 480 system with pre-installed LightCycler 480 Software. Western blot images were collected using Bio-Rad ChemiDoc Imaging System with Image Lab Software. Microscopy and Histology images were collected using Carl Zeiss LSM 880 and Ni-U Upright microscope. Absorbance, fluorescence, and luminescence data were collected using SpectraMax iD3 multi-mode microplate reader. Animal imaging was acquired using Xenogen IVIS2000 In Vivo Imaging System. RNA-seq and Chip-seq data were collected using Illumina HiSeq1500
Data analysis	The experimental data was analyzed using the following software: GraphPad Prism v8, FlowJo v10, ZEN 2.3 blue edition, Bio-Rad Image Lab 6.0.1, Photoshop CS6, QuPath 0.2.3, ImageJ (1.52a), IGV (2.7.2), Histat2 (2.1.0), StringTie (1.3.6), Samtools (1.7), R (v4.1.0), DEseq2 (1.32.0), clusterProfiler (4.0.5).

For manuscripts utilizing custom algorithms or software that are central to the research but not yet described in published literature, software must be made available to editors and reviewers. We strongly encourage code deposition in a community repository (e.g. GitHub). See the Nature Portfolio guidelines for submitting code & software for further information.

Data

Policy information about availability of data

All manuscripts must include a data availability statement. This statement should provide the following information, where applicable:

- Accession codes, unique identifiers, or web links for publicly available datasets
- A description of any restrictions on data availability
- For clinical datasets or third party data, please ensure that the statement adheres to our policy

The raw data in the fastq files of the RNA-sequencing and ChIP-sequencing have been deposited in Sequence Read Archive (RA) on National Center for Biotechnology Information (NCBI)) under accession number PRJNA1007461 [https://www.ncbi.nlm.nih.gov/bioproject/PRJNA1007461]. All remaining data can be found in the Article, Supplementary and Source data files."

Human research participants

Policy information about studies involving human research participants and Sex and Gender in Research.

Reporting on sex and gender	The NPC PDX Xeno-76 was established in our previous report study (Lin et al, Establishment and characterization of new tumor xenografts and cancer cell lines from EBV-positive nasopharyngeal carcinoma.Nat Commun. 2018 Nov 7;9(1):4663. doi: 10.1038/s41467-018-06889-5.). The stably Xeno-76 PDX was derived from the NPC tumor specimen of a female patient.
Population characteristics	The NPC PDX Xeno-76 was derived from the undifferentiated NPC of a Chinese patient in Hong Kong. No patient data analysis was conducted in this study.
Recruitment	One NPC PDX Xeno-76 is used in this study.
Ethics oversight	The collection and use of these NPC specimens for the study of PDX establishment study were approved by the Institutional Review Board of the University of Hong Kong, and the patients' consents were obtained (Nat Commun. 2018 Nov 7;9(1):4663. doi: 10.1038/s41467-018-06889-5.).

Note that full information on the approval of the study protocol must also be provided in the manuscript.

Field-specific reporting

Please select the one below that is the best fit for your research. If you are not sure, read the appropriate sections before making your selection.

☒ Life sciences ☐ Behavioural & social sciences ☐ Ecological, evolutionary & environmental sciences

For a reference copy of the document with all sections, see nature.com/documents/nr-reporting-summary-flat.pdf

Life sciences study design

All studies must disclose on these points even when the disclosure is negative.

Sample size	Sample size was determined to ensure statistical analyses. No statistical methods were used to predetermine sample size. For the in vivo experiments, 6-8 mice per group of treatment were used. Sample sized were chosen based in our previous study (Bruce et al. Nat Commun. 2021, 12:4193).
Data exclusions	No data were excluded.
Replication	All experiments were conducted at least 3 independent times with similar results if not otherwise specified. Therefore, all attempts at replication were successful.
Randomization	The experiments were randomized for the treatment efficacy studies in mice. Mouse allocation was random in the in vivo studies. All other experiments, subjects were randomly assigned to experimental groups.
Blinding	For IHC and RNAscope ISH quantification analysis, a NPC pathologist was blinded to treatment groups and performed quantification for the staining. Blinding was not possible for other experiments as the investigators must be aware of what treatment to give cells and animals.

Reporting for specific materials, systems and methods

We require information from authors about some types of materials, experimental systems and methods used in many studies. Here, indicate whether each material, system or method listed is relevant to your study. If you are not sure if a list item applies to your research, read the appropriate section before selecting a response.

Materials & experimental systems

n/a	Involved in the study
☐	☒ Antibodies
☐	☒ Eukaryotic cell lines
☒	☐ Palaeontology and archaeology
☐	☒ Animals and other organisms
☒	☐ Clinical data
☒	☐ Dual use research of concern

Methods

n/a	Involved in the study
☐	☒ ChIP-seq
☐	☒ Flow cytometry
☒	☐ MRI-based neuroimaging

Antibodies

Antibodies used

anti-BZLF1/Zta (BZ1, Santa Cruz Biotechnology, Dallas, TX, USA; 1:1000 or 1:100); anti-Rta (8C12, Argene, Varilhes, France;1:1000); anti-EA-D (1108-1, Santa Cruz Biotechnology; 1:1000; or 1:100); anti-VCAp18 (#PA1-73003, Invitrogen; 1:500); anti-cleaved caspase 3 (Asp175, Cell Signaling; 1:1000); anti-Actin (13E5, Cell Signaling; 1:4000); anti-gp350 (0221, Santa Cruz Biotechnology; 1:100) antibodies and EBV BGLF4 monoclonal antibody (MAb) 2616 (1:1000; generated by Chen MR; Ref: Wang J.T. et. al. J Gen Virol 86:3215–3225 (2005)) were used in Western Blotting and IHC experiments. Alexa-647-conjugated mouse anti-BZLF1/Zta antibody (BZ1, Santa Cruz Biotechnology; 1:100), Alexa-594-conjugated anti-EA-D antibody (1108-1, Santa Cruz Biotechnology; 1:100), Alexa-488-conjugated anti-gp350 antibody (Santa Cruz Biotechnology; 1:100) were used for FACS and IF experiments. Anti-FLAG antibody M2 (F1804, Sigma) was used for ChIP-sequencing experiments.

Validation

All antibodies in the study were used according to the user manuals and validation statements can be found on the respective manufacture websites or published reports.
anti-BZLF1/Zta antibody (BZ1, Santa Cruz Biotechnology): https://www.scbt.com/p/ebv-zebra-antibody-bz1;
anti-Rta antibody (8C12, Argene, Varilhes, France): was validated for western blotting in previous reports (Yiu. S.P. et al. Cancers. 10:505 (2018); Hui, K.F. et al. Int J Cancer. 138:125-36 (2016));
anti-EA-D antibody (1108-1, Santa Cruz Biotechnology): https://www.scbt.com/p/ebv-ea-d-antibody-1108-1;
anti-VCAp18 antibody (#PA1-73003, Invitrogen): https://www.thermofisher.com/antibody/product/Epstein-Barr-Virus-p18-Antibody-Polyclonal/PA1-73003;
anti-cleaved caspase 3 antibody (Asp175, Cell Signaling): https://www.cellsignal.com/products/primary-antibodies/cleaved-caspase-3-asp175-antibody/9661;
anti-Actin antibody (13E5, Cell Signaling; 1:4000): https://www.cellsignal.com/products/primary-antibodies/b-actin-13e5-rabbit-mab/4970;
anti-gp350 antibody (0221, Santa Cruz Biotechnology; 1:100): https://www.scbt.com/p/ebv-gp350-envelope-protein-antibody-0221;
EBV BGLF4 monoclonal antibody (MAb) 2616 : was validated for western blotting in previous report (Wang J.T. et. al. J Gen Virol 86:3215–3225 (2005));
anti-FLAG antibody M2 (F1804, Sigma): https://www.sigmaaldrich.com/HK/en/product/sigma/f1804 .

Eukaryotic cell lines

Policy information about cell lines and Sex and Gender in Research

Cell line source(s)

The EBV-positive cell lines C666-1, C17, NPC43, NPC43-M81, NPC76c and EBV-negative NPC cell line HK1 were established in our laboratory as described in the published reports (Cheung, S.T., et al. Int J Cancer. 83, 121-6 (1999); Yip, Y. L. et al. Lab Invest. 98, 1093-104 (2018); Lin, W. et al. Nat Commun. 9, 4663 (2018); Huang, D.P. et al. Int J Cancer. 26, 127-32 (1980)). The EBV-positive gastric cancer cell lines, YCCEL1 and AGS-EBV were provided by Professors Qian Tao (Kim, D.H., et. al. J Gen Virol. 94, 497-506 (2013) and Jun Yu (Liang, Q., et al. Gastroenterology. 147, 1350-62 (2014) respectively. The EBV-positive Akata cell line Akata-EBV was provided by Professor Kenzo Takada (Takada, K. et al. Virus Genes. 5:147-56 (1991). EBVaGC cell line SNU719 was obtained from the Korean Cell Line Bank, Seoul, Republic of Korea. (KCLB No: 00719.1). The EBV-positive BL cell line P3HR-1 was obtained from ATCC (HTB-62).

Authentication

All of the cell lines and patient derived xenografts have been authenticated by STR profiling. The EBV status in these cell lines has been verified by EBER in-situ hybridization. The tests were performed before we used the cell lines for the experiments and on 8th and 9th January 2024.

Mycoplasma contamination

All the cell lines have been tested and confirmed to be negative for mycoplasma contamination.

Commonly misidentified lines
(See ICLAC register)

None.

Animals and other research organisms

Policy information about studies involving animals; ARRIVE guidelines recommended for reporting animal research, and Sex and Gender in Research

Laboratory animals

5-6 weeks old NOD/SCID mice

Wild animals	No wild animals were used in the study.
Reporting on sex	Female
Field-collected samples	No field-collected samples were used in the study.
Ethics oversight	All animal care and experimental procedures were approved by the University Animal Experimentation Ethics Committe (AEEC), The Chinese University of Hong Kong. The animal license was obtained from the Hong Kong SAR Government, Department of Health.

Note that full information on the approval of the study protocol must also be provided in the manuscript.

ChIP-seq

Data deposition

☒ Confirm that both raw and final processed data have been deposited in a public database such as GEO.

☒ Confirm that you have deposited or provided access to graph files (e.g. BED files) for the called peaks.

Data access links<br>*May remain private before publication.*	We have deposited the raw RNA and ChIP-sequencing file to the Sequence Read Archive (SRA) on NCBI;<br>Accession number: PRJNA1007461.
Files in database submission	PRJNA1007461 EBV-associated epithelial cancer therapy Jan 15 '24
Genome browser session<br>(e.g. UCSC)	https://www.ncbi.nlm.nih.gov/bioproject/PRJNA1007461

Methodology

Replicates	Multiple biological replicates are included.
Sequencing depth	Sequenced to 10M total raw reads, paired-end mode,150 read length, over 97% of uniquely mapped reads
Antibodies	Anti-FLAG (F1804, Sigma)
Peak calling parameters	MASC2 callpeak function with -q0.01
Data quality	FastQC was used to check the raw data sequencing
Software	fastqc (0.12.1), bowtie2 (2.3.4.3), picard (22.3.1), MACS2 (2.2.4), diffBind (3.2)

Flow Cytometry

Plots

Confirm that:

☒ The axis labels state the marker and fluorochrome used (e.g. CD4-FITC).

☒ The axis scales are clearly visible. Include numbers along axes only for bottom left plot of group (a 'group' is an analysis of identical markers).

☒ All plots are contour plots with outliers or pseudocolor plots.

☒ A numerical value for number of cells or percentage (with statistics) is provided.

Methodology

Sample preparation	For BZLF1 positive cell detection, tumor cells were collected after treatment and fixed with 4% PFA at room temperature for 15 min followed by 30 min permeabilization. Then cells were stain ed with Zta-Alexa647 for 2 hours in dark at room temperature. For cell cycle analysis, tumor cells were fixed with ice cold 75% ethanol in -20 degree C for at least 2 hours. Cells were then stained with PI in dark for 15min.
Instrument	BD LSR Fortessa
Software	FACSDiva for acquisition; FlowJo v10 for analysis
Cell population abundance	No cell sorting was performed in this study.
Gating strategy	Cells were gated based on FSC/SSC parameters, and then the debris and doublet were exclusion based on the FSC-W/FSC-A parameters. Untreated sample was used as negative control.

☒ Tick this box to confirm that a figure exemplifying the gating strategy is provided in the Supplementary Information.
